# Machine Learning Techniques to Predict Mental Health Diagnoses: A Systematic Literature Review

**DOI:** 10.2174/0117450179315688240607052117

**Published:** 2024-07-26

**Authors:** Ujunwa Madububambachu, Augustine Ukpebor, Urenna Ihezue

**Affiliations:** 1 School of Computing Sciences and Computer Engineering, University of Southern Mississippi, Hattiesburg, Mississippi, United States of America; 2 Cyber Insights, Jackson, Mississippi, United States of America; 3 Department of Public Health, College of Nursing and Health Professions, University of Southern Mississippi, Hattiesburg Mississippi, United States of America

**Keywords:** Mental health, Machine learning, Prediction, Algorithm, Deep learning, CNN

## Abstract

**Introduction:**

This study aims to investigate the potential of machine learning in predicting mental health conditions among college students by analyzing existing literature on mental health diagnoses using various machine learning algorithms.

**Methods:**

The research employed a systematic literature review methodology to investigate the application of deep learning techniques in predicting mental health diagnoses among students from 2011 to 2024. The search strategy involved key terms, such as “deep learning,” “mental health,” and related terms, conducted on reputable repositories like IEEE, Xplore, ScienceDirect, SpringerLink, PLOS, and Elsevier. Papers published between January, 2011, and May, 2024, specifically focusing on deep learning models for mental health diagnoses, were considered. The selection process adhered to PRISMA guidelines and resulted in 30 relevant studies.

**Results:**

The study highlights Convolutional Neural Networks (CNN), Random Forest (RF), Support Vector Machine (SVM), Deep Neural Networks, and Extreme Learning Machine (ELM) as prominent models for predicting mental health conditions. Among these, CNN demonstrated exceptional accuracy compared to other models in diagnosing bipolar disorder. However, challenges persist, including the need for more extensive and diverse datasets, consideration of heterogeneity in mental health condition, and inclusion of longitudinal data to capture temporal dynamics.

**Conclusion:**

This study offers valuable insights into the potential and challenges of machine learning in predicting mental health conditions among college students. While deep learning models like CNN show promise, addressing data limitations and incorporating temporal dynamics are crucial for further advancements.

## 1 INTRODUCTION

In recent times, the utilization of Machine Learning (ML) techniques for predicting mental health diagnoses among students has gained significant traction. The rise in mental health issues among student populations has prompted a pressing concern for educators, healthcare professionals, and policymakers. Mental health profoundly impacts emotions, reasoning, and social interactions, necessitating innovative prevention and intervention strategies, especially for college students. Early detection is crucial, and medical predictive analytics could revolutionize healthcare, particularly with the profound impact of mental health on individuals.

Machine learning techniques, which fulfill the purposes of data analysis, prediction, and deriving meaning from data, have become invaluable for predicting mental health. Two main types of ML, namely supervised learning and unsupervised learning, are commonly employed in mental health research [1]. Supervised learning, utilizing structured training data, is extensively used in medical research, while the application of unsupervised learning in clinical settings is limited [2]. Reinforcement Learning (RL) is not covered in this paper due to its limited relevance to mental health data, as it focuses on agents learning optimal behaviors in interactive environments.

Over time, collected information undergoes processing and analysis using various machine learning techniques to enhance platform usability and develop interactive tools. Machine learning, a part of Artificial Intelligence (AI), aims to impart knowledge to computers by leveraging data, observations, and real-world interactions [3]. The availability of abundant data, cost-effective storage, and powerful computational systems has propelled machine learning, elevating it from a mere pattern recognition algorithm to encompass Deep Learning (DL) approaches. Liu *et al*. examined depression among college students, highlighting its detrimental effects on health, academics, and social life.

The study reviews factors contributing to depression, predictive methods, and non-pharmaceutical interventions [4]. Another significant study by Johnson *et al*. applied unsupervised learning to identify patterns linked to anxiety and stress among university students, analyzing physiological and behavioral data collected from wearable devices [5].

Kirlic *et al*. explored machine learning algorithms, including logistic regression, decision trees, random forests, and deep learning techniques for early detection of suicidal tendencies in college students, using data from student counseling centers and campus resources [6]. Integrated machine learning techniques with electronic health records to predict the likelihood of mental health issues among college students showcase the potential for identifying risk factors and tailoring personalized interventions [7, 8].

Sofianita Mutalib *et al*. specifically employed Decision Tree, Neural Network, Support Vector Machine, Naive Bayes, and logistic regression algorithms to categorize students based on different mental health problems, revealing distinct optimal models for specific concerns [9]. Ashley E. Tate carried out a comparative analysis of various machine learning techniques, indicating the superior performance of the random forest model in mental health prediction [10].

Machine Learning (ML) has emerged as a valuable tool in understanding and addressing mental health issues [11]. Its application in mental health diagnosis demonstrates the potential for ML algorithms to analyze vast amounts of data, identify patterns, and provide valuable insights into various disorders. Fried *et al*. introduced the possibility of using Deep Learning (DL) methods not only to predict and diagnose specific mental health disorders but also to simultaneously identify comorbidities and interconnected conditions [12]. The intricate neural network architectures of deep learning models enable them to capture complex relationships within the data, offering a more comprehensive understanding of the multifaceted nature of mental health.

Despite the success of machine learning algorithms, there is a lack of explicit justification by many researchers for their chosen ML methods, raising concerns about potential oversights in leveraging algorithmic strengths for specific mental health analysis tasks [1]. Additionally, a worrisome trend of applying ML algorithms without a thorough understanding of the underlying data characteristics has been noted, compromising the reliability and generalizability of study findings. This emphasizes the critical need for researchers to consider the compatibility between the selected ML algorithm and the nuances of the mental health data under investigation.

### 1.1 Contributions of the Study

Despite the promising potential of machine learning in predicting mental health diagnoses in college students, several limitations and challenges persist. One primary concern is the lack of standardized, high-quality datasets that adequately represent the diversity and complexity of mental health conditions. Ethical considerations, such as data privacy and potential biases in the training data, are critical problems that must be addressed to ensure the fair use of machine learning models. Another challenge lies in the interpretability of complex models like deep neural networks, which can hinder understanding of how decisions are made. Furthermore, mental health stigma can affect data collection and introduce biases in self-reported information, leading to skewed results. Consequently, this comprehensive review contributes to this emerging research area and aims to:

#### 1.1.1 Identify the Datasets Used for the Prediction of Mental Health Diagnoses

This study identified high-quality datasets for developing robust and generalizable predictive models, enhancing the accuracy and applicability of the results in real-world scenarios.

#### 1.1.2 Uncover Methodologies and Key Findings and Identify Commonly Used Algorithms in Mental Health Diagnosis Prediction

This research contributes to understanding methodologies, key findings, and trends in using machine learning to predict mental health diagnoses. Through an extensive literature review, it identifies the latest advancements and emerging patterns in the field. Identifying popular techniques will help researchers select appropriate models for their specific research objectives.

#### 1.1.3 Assess the Effectiveness and Accuracy of Machine Learning Models

This research evaluates machine learning model performance in predicting mental health diagnoses. Examining previous research outcomes establishes the effectiveness and accuracy of these models in various contexts. It also reveals the strengths and weaknesses of different approaches, guiding researchers in making informed decisions about selecting and optimizing predictive models.

## 2 REVIEW BACKGROUND

Reports by the World Health Organization (WHO) emphasize the critical role of mental health in overall well-being, recognizing it as a fundamental human right existing on a continuum from optimal well-being to severe suffering [13].

The COVID-19 pandemic has significantly impacted global mental health, increased rates of anxiety and depression, and widened the treatment gap. Anxiety and depression are prevalent worldwide, with suicide being a leading cause of mortality, particularly among young individuals. Moreover, severe mental health conditions often lead to premature mortality due to preventable physical illnesses. Despite these challenges, global mental health systems face substantial gaps and disparities in information, research, governance, resources, and services. To address these issues, this research conducted a literature review following a structured eight-step approach proposed by Okoli [14], ensuring scientific rigor throughout the process.

Fig. (**1**) explains the systematic review of machine learning techniques for predicting mental health diagnoses following a rigorous eight-step methodology. Firstly, a clear research question was defined. A comprehensive search strategy was then developed, including database selection and search term formulation. Studies retrieved were screened based on predefined criteria. Selected studies underwent effectiveness assessment, considering methodological aspects. Pertinent data were systematically extracted using a standardized form. A comprehensive analysis followed, identifying patterns and themes across studies. Depending on the research question, either quantitative (meta-analysis) or qualitative (thematic analysis) synthesis was conducted. Finally, findings were summarized, including re findings were summarized, including results, conclusions, and implications, in a systematic review report or academic publication. By adhering to these steps, this research ensured a systematic, rigorous, and comprehensive approach to collecting and analyzing relevant evidence.

Fig. (**2**) categorizes mental illness diagnoses, including bipolar disorder, schizophrenia, PTSD, depression, anxiety, and ADHD. It also organizes machine learning approaches into supervised learning, unsupervised learning, neural networks, and deep learning based on their respective learning methods. Furthermore, this study assesses the performance of these models to illustrate their effectiveness in mental health applications.

### 2.1 Bipolar Disorders

Bipolar disorders are a group of serious and long-lasting mental health conditions [15-17]. There are two main types: bipolar I disorder, which involves experiencing manic episodes, and bipolar II disorder, which involves hypomanic episodes and major depressive episodes. These disorders have a significant impact on how well a person can do everyday tasks and are associated with a reduced lifespan of about 10 to 20 years.

### 2.2 Schizophrenia

Schizophrenia, a multidimensional mental health illness, presents long-term issues for both people and families. It usually appears early in childhood and causes symptoms, such as skewed beliefs, sensory experiences disconnected from reality, and issues with cognitive functioning [18-20]. These symptoms remain over time, limiting the affected person's capacity to participate in everyday activities and maintain social interactions.

Negative symptoms of Schizophrenia, which include difficulties in emotional expression and motivation, as well as cognitive impairments, such as attention deficits and reduced executive function, exacerbate the handicap experienced by persons suffering from the disorder [21]. Schizophrenia is a common mental condition that affects around 1% of the world's population, with diagnostic criteria established in the Diagnostic and Statistical Manual of Mental Disorders (DSM-5) [21]. This systematic guide supports physicians in diagnosing and treating persons, showing the hallmark signs of schizophrenia, allowing for appropriate interventions and support for those afflicted by this difficult disorder.

### 2.3 Post-traumatic Stress Disorder

The DSM-5 [21] delineates diagnostic criteria for PTSD, requiring exposure to potentially life-threatening events accompanied by specific symptoms persisting for at least a month, causing distress or impairment. Risk-taking behaviors encompass actions with uncertain outcomes, such as substance abuse, delinquency, poor health, unhealthy eating, and unprotected sex [22-24]. Studies highlight a correlation between exposure to trauma, the development of PTSD, and subsequent engagement in risk-taking behaviors [25-28]. Childhood maltreatment predicts higher levels of risky behavior in adolescence and adulthood, with sexual abuse being a significant factor [29]. These findings underscore the interplay between traumatic experiences, mental health, and behavioral outcomes.

### 2.4 Depression and Anxiety

Depression, clinically known as major depressive disorder, is assessed using the Patient Health Questionnaire (PHQ) [30]. It is characterized by profound sadness and loss of interest, significantly affecting daily life. Shorey *et al*. found that 34% of adolescents aged 10-19 are at risk of clinical depression, exceeding estimates for those aged 18-25 [31].

Depression prevalence is highest in the Middle East, Africa, and Asia, with females more affected than males. Untreated depression can lead to suicidal thoughts [32]. Anxiety, defined by the APA, involves tension, worry, and physical symptoms like increased heart rate and muscle tension [33]. It arises from perceived threats or stressors, real or imagined.

### 2.5 Attention-deficit Hyperactivity Disorder

ADHD is a neurodevelopmental illness characterized by symptoms, such as inattention, hyperactivity, and impulsivity [34]. These symptoms frequently emerge in numerous facets of everyday living, providing difficulty for those with the illness. ADHD is not just a childhood disorder; it may last into adolescence and age, impacting people all their lives. Its ubiquity makes it one of the most widely diagnosed mental health problems, impairing people's ability to focus, manage their impulses, and engage successfully in daily activities.

## 3 METHODS

The systematic review followed the Preferred Reporting Items for Systematic Reviews and Meta-Analysis (PRISMA) method, recognized as the gold standard for structured, systematic reviews and meta-analyses. This method offers authors a comprehensive framework, facilitating a thorough examination of concepts discussed in scholarly articles across diverse research fields. A pivotal aspect of this methodology involves precisely defining eligibility criteria, which is crucial for formulating the research hypothesis. In line with PRISMA guidelines, the review included sections on search methodology, inclusion and exclusion criteria, and data extraction. Employing a PRISMA checklist, the review aimed to enhance the quality and precision of the evaluation process for all analyzed articles [14].

### 3.1 Search Strategy

The systematic review focused on assessing machine learning techniques for predicting mental health diagnoses. The search strategy encompassed keywords like “deep learning,” “mental health prediction,” and “mental health diagnoses” conducted across reputable repositories, such as IEEE Xplore, ScienceDirect, Pubmeb, and Elsevier, among others [8]. Only published papers specifically addressing machine learning and deep learning models for mental health diagnoses were considered, with duplicate papers eliminated.

### 3.2 Inclusion and Exclusion Criteria

**Fig. (3):**
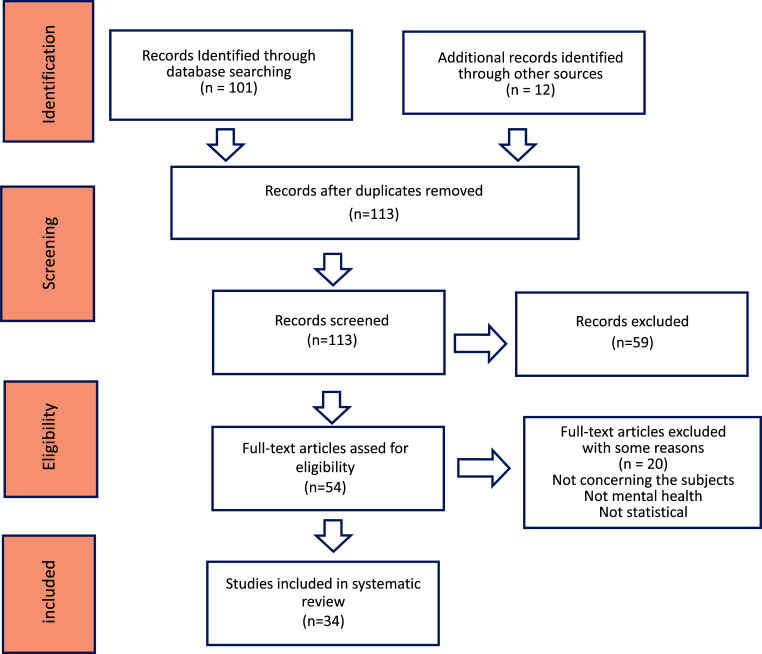
Flow diagram of the study selection process.

The study focused on reviewing papers published between 2011 and 2024, emphasizing deep learning models for mental health diagnoses. Initially, 101 articles were identified, with 12 more found through alternative methods. After screening, 30 relevant studies were included for evaluation (Fig. **3**). There were no restrictions on machine learning algorithms, study country, language (English), or population demographics. Mental health conditions of interest included bipolar disorder, ADHD, schizophrenia, PTSD, depression, and anxiety. Duplicate publications were rigorously removed following the PRISMA flowchart for transparency. Studies failing to meet at least two performance criteria, as well as newspapers, magazines, proposals, and posters, were excluded.

### 3.3 Data Extraction and Analysis

The chosen articles underwent comprehensive evaluation covering content, references, machine learning methodologies, performance metrics, and dataset origins, and identified limitations or areas for future investigation. Fig. (**4**) presents a graphical representation illustrating the distribution of articles included in the review spanning from 2011 to 2024. Notably, 2021 and 2022 exhibited the highest count, each featuring seven papers. In contrast, 2023 contributed four papers, while 2017 and 2020 each accounted for three. The years 2013, 2014, 2016, and 2018 had the lowest contribution, with one paper each. Interestingly, several two-year periods displayed identical numbers of papers, underscoring a consistent trend in research output throughout the years [8].

### 3.4 Data Synthesis and Presentation

This research article presents a comprehensive review of machine learning methods for predicting mental health diagnoses. Spanning the last 14 years, the study evaluates recent advancements in the field, employing a transparent methodology and search strategy to bolster reliability and replicability. Key findings are highlighted, with particular emphasis placed on the outcomes of interest, including Bipolar Disorders (6 papers), Schizophrenia Prediction (4 papers), PTSD (6 papers), Depression and Anxiety (10 papers), and ADHD (4 papers), totaling 30 selected papers detailed in Table **1**. Although the review lacks official registration, it benefits from non-financial support from academic institutions, peer reviewers, and research collaborators. No external funding is acquired, ensuring transparency and unbiased reporting.

## 4 RESULTS

The summary of datasets in Table **1** utilized in 30 studies included in this review spans a diverse array of sources, including data on male and female students covering grades 5 to 9, IBM^®^ MarketScan^®^ Commercial Subset, clinical data, resting-state functional magnetic resonance imaging (rsfMRI) data, Twitter data, and more. Dataset sizes ranged from 50 to 2,500,000 records, reflecting the variability and scale of the data sources utilized. The studies targeted a wide range of outcome variables, including near-term suicidal behaviors, diagnosis of Post-Traumatic Stress Disorder (PTSD), presence of Childbirth-related PTSD (CB-PTSD), suicide attempts, Bipolar Disorder (BD), Attention-Deficit/Hyperactivity Disorder (ADHD), depression, and anxiety. Prediction nature varied across studies, with aims such as predicting the likelihood of individuals engaging in suicidal behaviors, identifying PTSD patients from structured and unstructured medical records, and predicting PTSD diagnosis probability in firefighters exposed to trauma. Variable sources included surveys, experiments, observations, and existing databases, while variable types encompassed categorical, continuous, ordinal, and binary variables, highlighting the complexity and heterogeneity of mental health data.

The systematic review aimed to evaluate the performance of thirty classification algorithms in predicting five different diseases, particularly focusing on mental health. It encompassed advancements in machine learning algorithms from 2011 to 2024. Inclusion criteria involved scrutinizing research papers and employing a comprehensive search across databases. Measures, such as eliminating duplicates and adhering to the PRISMA flowchart, were implemented for reliability. The major evaluated classifiers included Random Forest, Logistic Regression, Support Vector Machine (SVM), Multi-layer Perceptron (MLP), Decision Tree, Naive Bayes, K-nearest neighbors, Gradient Boosting Machine (GBM), and Convolutional Neural Network (CNN).

### 4.1 Approaches for Bipolar Disorder Detection

Machine learning techniques have emerged as valuable tools for identifying and detecting bipolar disorder, a complex mental illness characterized by extreme mood swings. Timely diagnosis is crucial for effective management. Birner *et al*. examined how LR can aid in diagnosing bipolar disorder, aiming to decrease misdiagnosis rates and shorten diagnosis time [55]. Sonkurt *et al*. developed a prediction algorithm utilizing CANTAB neurocognitive battery and a novel machine-learning approach to differentiate bipolar disorder patients from healthy controls, achieving a 78% accuracy rate [56]. Passos *et al*. identified a suicidality signature among mood disorder patients, including bipolar disorder, using machine learning [57]. Chen *et al*. presented a support vector machine (SVM) for detecting brain structural changes as biomarkers from magnetic resonance images. The SVM demonstrates superior performance in bipolar disorder datasets, achieving an AUC of 80.6%. It offers the potential for automatic diagnosis and mechanism studies in neurological and psychiatric diseases [58]. These studies underscore the potential of machine learning to enhance early detection, diagnostic precision, and personalized treatment strategies for bipolar disorder.

### 4.2 Approaches for Schizophrenia Prediction

Several studies have showcased the effectiveness of machine learning (ML) techniques in predicting schizophrenia. While Bohaterewicz *et al*. concentrated on leveraging machine learning and advanced neuroimaging to improve prediction of suicide risk in schizophrenic patients [38], Kirchebner *et al*. employed Boosted Classification Trees to explore the factors influencing violent behavior in the same population [39]. This dual approach highlights the potential of machine learning for both improving risk prediction of suicide and identifying factors associated with violence in schizophrenia, paving the way for better patient outcomes and targeted interventions. Hahn *et al*. achieved an impressive 84% accuracy using Support Vector Machine (SVM) and diffusion tensor imaging data [59]. Building on prior research, Hettige *et al*. and Birnbaum *et al*. explored machine learning for mental health diagnosis using different algorithms, like SVM, LR, RF, *etc* [60, 61]. Notably, Birnbaum *et al*. performed better in identifying schizophrenia *via* social media analysis [61]. Similarly, Hettige *et al*. focused on developing models to predict suicide attempts among individuals already diagnosed with schizophrenia spectrum disorders [60].

In summary, ML shows promise in schizophrenia prediction, especially when utilizing neuroimaging and genetic data in multimodal approaches. Overcoming challenges like sample sizes and embracing longitudinal research could advance the early detection and management of schizophrenia.

### 4.3 Approaches for Post-traumatic Stress Disorder Detection

Various machine learning techniques have been explored for detecting Post-Traumatic Stress Disorder (PTSD), leveraging diverse datasets and methodologies. Costa *et al*. proposed Support Vector Machines (SVM) using physiological signals [64], while Banerjee *et al*. focused on Long Short-Term Memory (LSTM) neural networks with textual features [65]. He *et al*. combined Random Forest, AdaBoost, and SVM with demographic and behavioral features [66]. Lekkas *et al*. explored the use of GPS data from smartphones to detect PTSD diagnostic status among previously traumatized women, achieving high predictive performance with an AUC of 0.816, balanced sensitivity of 0.743, balanced specificity of 0.8, and balanced accuracy of 0.771, suggesting the potential utility of GPS information as a digital biomarker for PTSD [67], Beymohammadi *et al*. used Convolutional Neural Networks (CNN) with EEG signals [68], and Miotto *et al*. utilized Deep Learning models with electronic health records [69]. Jeffrey *et al*. employed machine learning on social media data for PTSD signs [70]. These studies collectively illustrate diverse methodologies and data sources, contributing to a comprehensive understanding of PTSD detection. Despite limitations, this body of research highlights the potential of machine learning in aiding PTSD detection and advancing treatment strategies.

### 4.4 Approaches for Depression and Anxiety Detection

Recent studies have leveraged machine learning (ML) techniques to predict mental health conditions, such as depression and anxiety. Chen *et al*. [71] developed a diagnostic model for adult ADHD. They demonstrated promising statistical accuracy, suggesting the potential of machine learning models, such as (SVM) and KNN, to inform clinical practice in diagnosing ADHD. Ojo *et al*. [72] employed Natural Language Processing (NLP) and sentiment analysis on social media data for depression detection. Alghowinem *et al*. [73] differentiated depressed individuals from controls using Gaussian Mixture Models (GMM) and Mel Frequency Cepstral Coefficients (MFCC) from speech data.

Watts *et al*. [74] utilized algorithms like Random Forests and SVM on EEG data to predict major depressive disorder (MDD) diagnosis. Deep learning methods, including Long Short-Term Memory (LSTM) networks and Convolutional Neural Networks (CNN), were applied by Chiong *et al*. [75] for anxiety and depression detection from social media texts. Yoon *et al*. and Xezonaki *et al*. [76, 77] contributed to depression detection using Multimodal Vlog Dataset and Hierarchical Attention Networks. These studies underscore the potential of ML in mental health screening and intervention.

### 4.5 Approaches for Attention-deficit/hyperactivity Disorder Detection

ADHD, a neurodevelopmental disorder characterized by symptoms like inattentiveness, hyperactivity, and impulsivity, necessitates early and accurate detection for effective management. Sinan *et al*. [78] proposed a method employing Convolutional Neural Networks (CNN) with multimodal feature fusion using resting-state functional MRI (rs-fMRI) and EEG data for precise ADHD classification. Shoeibi *et al*. [79] introduced a 3D CNN-based framework for rs-fMRI analysis, showing promising results in automatic ADHD diagnosis. Gurcan *et al*. [80] utilized Deep CNNs on functional near-infrared spectroscopy (fNIRS) data, achieving high accuracy in distinguishing ADHD patients. Arbabshirani *et al*. [81] integrated machine learning algorithms with structural and functional brain scans for individualized ADHD prediction. As mentioned in Table **1**, results demonstrated that DT [71] outperformed other algorithms in predicting ADHD from images with an accuracy of 86.6%. This suggests that DT has slightly superior performance in ADHD prediction using provided images compared to other classification models.

## 5 DISCUSSION

The increasing prevalence of mental health disorders, coupled with the advancement of technology, has led to a growing interest in utilizing machine learning techniques for early detection and diagnosis. In recent years, the potential of machine learning in detecting a range of mental health disorders, including bipolar disorder, schizophrenia, PTSD, depression, and anxiety, has gained significant attention. These disorders pose a substantial challenge to mental healthcare due to their complex nature and the limitations of traditional diagnostic methods.

This review delves into a collection of studies that have explored the application of machine learning in detecting mental health disorders. These studies showcase the promise of machine learning approaches in improving the accuracy and efficiency of diagnosis. However, it is crucial to critically evaluate both the strengths and limitations of these studies to gain a comprehensive understanding of their implications.

Liu *et al*. and Johnson *et al*. [4, 5] employed natural language processing to anticipate bipolar disorder using textual data and demonstrated the potential of neuroimaging data in differentiating bipolar disorder patients from healthy controls. For instance, in the context of mental health disorders, studies conducted by López Steinmetz *et al*. and Joshi *et al*. [45, 46] highlight the extensive assessment of the COVID-19 mental health implications among Argentine college students, as well as the creative application of Artificial Intelligence and Machine Learning algorithms for diagnosing depression and emotional states. Birner *et al*. [55] proposed that because of the variety of symptoms associated with bipolar disorder, correctly diagnosing bipolar disorder can take over nine years. Individuals with bipolar illness may benefit from early detection and care, which might dramatically enhance their quality of life and lifespan. The hypothesis is that machine learning approaches can help with the diagnostic process, potentially lowering misdiagnosis rates.

Transitioning to schizophrenia, Hahn *et al*. [59] showcased the power of neuroimaging data and support vector machines in achieving high accuracy in predicting schizophrenia. Hettige *et al*. [60] highlighted the serious problem of suicide among those suffering from schizophrenia, as well as the difficulty in recognizing those who are most likely to attempt suicide in the future. It emphasizes the ability of machine learning algorithms to include various risk variables and predict suicide attempts. However, it highlights the present ambiguity about how to effectively combine previously established risk variables into a useful prediction tool for evaluating the likelihood of suicide attempts in schizophrenia patients. Birnbaum *et al*. [61] reported that previous research demonstrated that language analysis of publicly available Twitter feeds may be used to discriminate persons who self-identify as having schizophrenia from healthy individuals. However, there have been few initiatives, including professional involvement, to examine the legitimacy of these diagnostic self-disclosures. The integration of multiple modalities, including clinical assessments, neuroimaging, and genetic information, demonstrated improved prediction accuracy and a better understanding of the heterogeneous nature of schizophrenia in studies by Bartal *et al*. [62] and Kim [63]. These articles explore innovative approaches to address mental health challenges; the first investigates using computational methods to screen for childbirth-related posttraumatic stress disorder, while the second focuses on developing an analysis model, leveraging AI algorithms and big data, to understand the prevalence of post-traumatic stress disorder among firefighters. However, sample size limitations and the dynamic nature of schizophrenia's progression pose challenges that need addressing.

In the case of PTSD, diverse approaches using physiological signals, textual features, EEG signals, and social media data have shown the potential of machine learning in detection. Costa *et al*. [30] utilized physiological signals, Banerjee *et al*. [65] focused on textual features, and Coppersmith *et al*., Beymohammadi **et al*.,* and Miotto *et al*. [67-69] analyzed EEG signals by employing natural language processing and deep learning models, respectively, on various data sources, revealing the versatility of machine learning in assisting with PTSD detection.

In the realm of depression and anxiety, studies explored audio/visual features, social media data, speech data, and EEG data to detect these conditions [72-74]. The application of deep learning models trained on social media texts by Chiong *et al*. [75] further underlines the potential of machine learning in this domain. However, the limitations encompassing small sample sizes and the necessity for validation hinder the full realization of their potential.

In summary, this review sheds light on the potential of machine learning in detecting mental health disorders, such as bipolar disorder, schizophrenia, PTSD, depression, and anxiety. The use of machine learning models presents avenues for early detection and personalized interventions, promising to enhance patient outcomes. Nevertheless, researchers must acknowledge the limitations within these studies, including small sample sizes, diverse datasets, and ethical considerations. Addressing these challenges is crucial for further validation and the eventual implementation of machine-learning approaches in mental health diagnostics (Table **S1**).

## CONCLUSION

This comprehensive study delves into the existing literature on the application of deep learning and machine learning techniques for predicting mental health outcomes, specifically among college students. The research demonstrates that these approaches exhibit promising potential in accurately diagnosing mental health conditions. Various algorithms and methods have been employed to analyze a range of data sources, including demographic data, clinical assessments, social media content, and neuroimaging data, effectively identifying individuals at risk of mental health disorders. Supervised learning methods, including Random Forest, Support Vector Machines (SVM), Extreme Learning Machine (ELM), as well as deep learning algorithms, such as Neural Network (NN) and Convolutional Neural Networks (CNN), have demonstrated effectiveness in forecasting mental health disorders.

Supervised learning methods, including Random Forest, Support Vector Machines (SVM), Extreme Learning Machine (ELM), as well as deep learning algorithms such as Neural Network (NN) and Convolutional Neural Networks (CNN), have demonstrated effectiveness in forecasting mental health disorders. Certain algorithms stood out using the author's dataset for each specific disease. Convolutional Neural Networks (CNNs) in bipolar disorder demonstrated outstanding performance, achieving an impressive accuracy rate and f-measure of 99.75%, surpassing other models. In the case of schizophrenia, SVM attained 90% for f-measure and 95% for AUC. For PTSD, both CNN and RF achieved a notable accuracy rate of 99%. In ADHD, ELM outperformed other algorithms with an AUC of 0.8757%. Lastly, neural networks showed the highest accuracy and AUC metrics of 99.03% for depression and anxiety.

However, challenges remain, including needing more extensive and diverse datasets, accounting for the diversity of mental health conditions, and integrating longitudinal data for temporal insight. Furthermore, improving the interpretability and transparency of machine learning models is crucial to fostering trust and acceptance in clinical settings. Despite these challenges, the application of machine learning in mental health prediction offers the potential for early detection, personalized interventions, and enhanced mental health outcomes among college students. Continuous research collaboration among researchers, clinicians, and policymakers is vital to fully harness the benefits of machine learning in mental health care.

## Figures and Tables

**Fig. (1):**
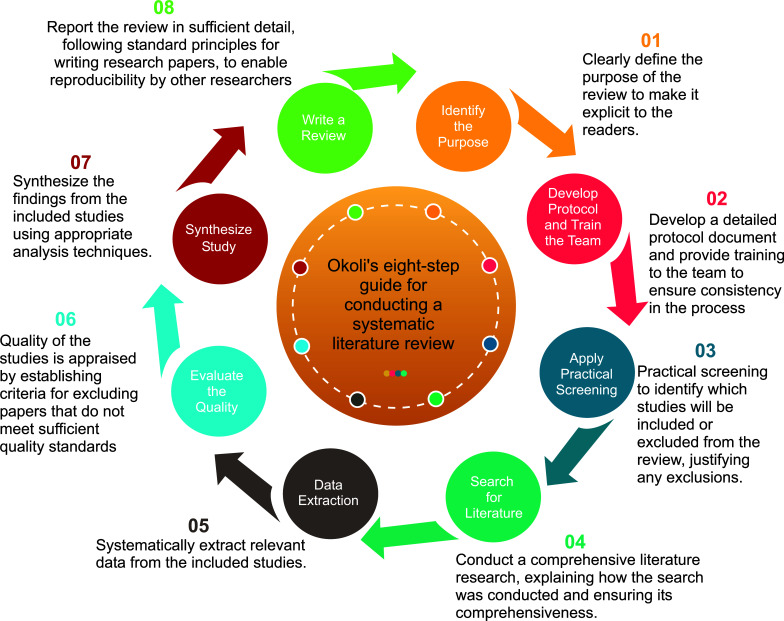
Okoli’s guide for conducting a standalone systematic literature review.

**Fig. (2):**
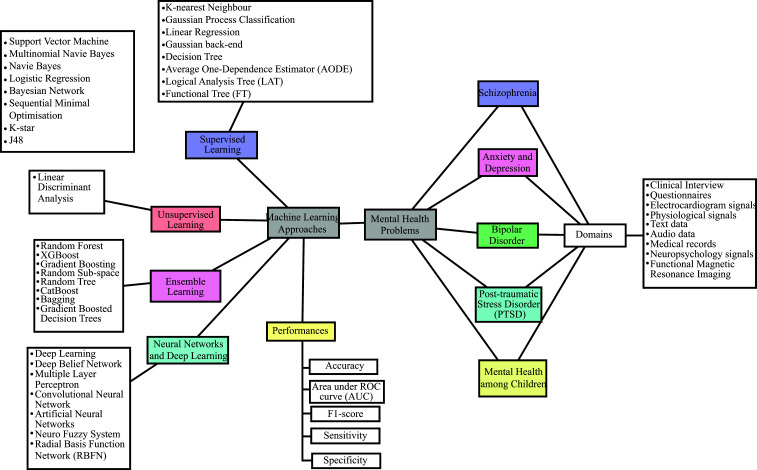
Taxonomy of the systematic literature review for this study [15].

**Fig. (4):**
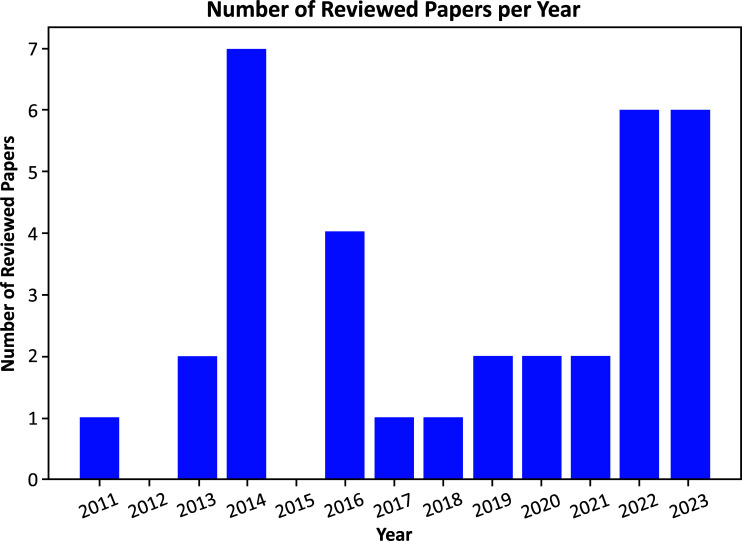
Diagram of reviewed papers.

**Table 1:** Overview of evaluation metrics for machine learning algorithms in mental disease prediction [35-82].

Paper	Disease	Dataset	Data Type	Sample size	Algorithm	Performance (%)			Motivation
				-		Accuracy	F-measure	AUC	
**Bipolar Disorder**									
[36]	BP	Clinical data, Hospital of Guangzhou Medical University	Images	80	SVM	87.5	-	0.939	The motivation stems from the challenges in accurately diagnosing BPD and the potential of neuroimaging techniques to provide valuable insights for improved diagnosis and treatment planning.
[37]	BP	sMRI	Images	212	CNN	99.72	99.75	99.75	To address the limitations of traditional machine learning techniques in effectively extracting deep information from neuroimaging data, which results in low classification accuracy of mental illnesses
[58]	BP	Public OASIS	Images	416	SVM	57	-	0.806	To develop a novel local feature-based support vector machine (SVM) approach that does not require deformation registration and is less influenced by artifacts, such as image distortion.
[35]	BP	Time series data of self-reported mood ratings using a bespoke smartphone app.	Text	50	SVM	80	-	0.86	To improve psychiatric diagnosis accuracy using mobile technology for real-time mood monitoring, employing a signature-based learning method to analyze mood data from individuals with bipolar disorder, borderline personality disorder, and healthy volunteers.
[55]	BP	BIPFAT dataset	Categorical data	341	LR	0.77	0.75	0.84	To leverage machine learning techniques to enhance the diagnostic process of bipolar disorder. The authors aim to reduce misdiagnosis rates and improve the overall quality of life for individuals with bipolar disorder.
[51]	BP	CANTAB	Categorical data	52	LASSO	0.78	0.80	0.78	To determine if neurocognitive deficits can distinguish bipolar disorder (BD) patients from healthy controls (HC).
**Schizophrenia**									
[38]	Schizophrenia	rsfMRI data	Image	59	LASSO	70	-	76	To bridge the gap between neuroscience and clinical practice by leveraging advanced imaging techniques and machine learning methods to enhance the understanding and prediction of suicidal risk in individuals with schizophrenia, potentially leading to improved patient outcomes and quality of life.
					LR	63	-	75	
					GB	65	-	74	
[39]	Schizophrenia	Zurich Centre for Inpatient Forensic Therapy Data	Text	370	Boosted Classification Trees	76.4	-	83	To investigate the factors that contribute to violent offending in individuals with schizophrenia spectrum disorders. Understanding these risk factors is crucial for developing effective preventive and therapeutic strategies to reduce the occurrence of violent behavior among this population.
[60]	Schizophrenia	Survey data	Text	345	LR	67	-	71	To develop machine learning models to predict suicide attempts among individuals diagnosed with schizophrenia spectrum disorders.
					RF	66	-	67	
					SVM	67	-	70	
					Elastic Net Model	65	-	71	
[61]	Schizophrenia	Twitter Data	Text	671	SVM	90	90	95	To enhance the accuracy of identifying individuals with schizophrenia based on their social media activity.
**PTSD**									
[40]	PTSD	Manitoba Primary Care Research Network (MaPCReN)	Text	154,118	CNN	99	71	86	To develop a predictive model that accurately identifies patients diagnosed with Post-Traumatic Stress Disorder (PTSD).
					RF	99	76	88	
[41]	PTSD	IBM® MarketScan® Commercial Subset	Text	44,342	RF	-	80	75	To identify and describe civilian patients who are likely to have undiagnosed post-traumatic stress disorder (PTSD) within the US commercial population.
[67]	PTSD	Global Positioning System (GPS) data	Text	185	Nested leave-one- subject-out (LOSO)	80.6	-	81.6	The study highlights the potential of mobile technology and passive sensing to advance the understanding and detection of PTSD outside traditional clinical settings, offering new avenues for research and development in this field.
[62]	PTSD	COVID-19 Data	Text	1,127	Deep Feedforward Neural Network (DFNN)	-	76	75	To analyze written narrative accounts of childbirth experiences. This approach aims to identify patterns in language use that may be indicative of childbirth CB- PTSD.
[63]	PTSD	Firefighter Data	Text	33,752	SVM	89	89	-	To address the prevalence of post-traumatic stress disorder (PTSD) among firefighters, who are often exposed to various traumatic events, such as accidents, disasters, and stress during the the course of their duties.
[82]	PTSD	Survey Data	Text	591	RF	72	-	88	To assess the impact of current and lifetime suicide ideation (SI) on the predictive validity of the Suicide Crisis Syndrome (SCS) for near-term suicidal behaviors (SB) using machine learning (ML) approaches.
**Attention-Deficit/Hyperactivity Disorder**									
[53] [54]	ADHD ADHD	fMRI ADHD 200 Global RsMRI, PKU, and NYU	Images Images	153	ELM	70	-	0.8757	To address the need for an effective, rapid, and objective diagnostic tool for attention-deficit/hyperactivity disorder (ADHD) to improve understanding, prevention, and treatment of the condition. To address the significant impact of attention deficit hyperactivity disorder (ADHD) on children, fill the gaps in understanding its pathophysiology, provide objective biological tools for diagnosis, and develop an efficient ADHD. Diagnosis method, and investigate altered executive functioning in ADHD.
					SVM- Linear	67.27	-	0.7792	
					SVM-RBF	66.36	-	0.8258	
				428	(XGBoost)	77	-	84.32	
[79]	ADHD	UCLA Dataset containing rsfMRI	Images	138	DT	60.90	64.22	-	To improve brain disorder diagnosis using fMRI data and deep learning.
					KNN	57.18	55.63	-	
					MLP	62.72	68.45	-	
					SVM	66.90	67.70	-	
					RF	62.72	63.61	-	
[71]	ADHD	NHS (SWYPFT)	Categorical data	69	SVM	72.46	-	0.784	This develops a diagnostic tool for ADHD using clinical data from NICE-compliant pathways to enhance clinician efficiency and accuracy, addressing the demand for reliable diagnosis amidst increasing awareness of the disorder.
					LR	72.46	-	0.795	
					DT	82.609	-	0.866	
					KNN	59.42	-	0.558	
					RF	81.159	-	0.866	
					NB	75.362	-	0.870	
**Depression and Anxiety**									
[42]	DA	Google Questionnaire	Categorical data	6,030	LR	9.897	-	98.89	Rising student mental health concerns necessitate early detection. This study utilizes Machine Learning to diagnose stress, anxiety, PTSD, ADHD, and depression, aiming to enhance prediction accuracy and mitigate adverse effects on academic performance and well-being. To develop an accurate diagnostic tool using resting-state fMRI (rs-fMRI) and a novel deep-learning approach to enhance the diagnosis of SZ and ADHD.
					RF	99.03	-	98.82	
					DT	92.58	-	92.87	
					KNN	98.57	-	98.98	
					NN	99.03	-	99.03	
[72]	DA	Reddit	Categorical data	2,809	BERT	0.924	0.924	-	
					CNN	0.6	0.4	-	
					LSTM	0.552	0.3562	-	
[43]	DA	R.G. Kar Medical College and Hospital, Kolkata Data	Categorical data	520	BN	79.8	79.7	88.9	To assist in predicting depression and anxiety in the life of the individual at an early stage.
					NB	79.6	79.4	85.3	
					LOG	72.4	72.2	81.1	
					MLP	77.8	77.8	85	
					SMO	75.3	74.6	75.9	
					KS	75.3	75.3	81.4	
					RS	87.5	87.5	91.7	
					J48	87.8	87.8	86	
					RF	89	89	94.3	
					RT	85.1	85.1	85	
[44]	DA	The American University of Beirut (AUB) and the Lebanese University (LU0 Survey)	Categorical data	329	MLP (D) (A)	-	68.42 68.29	73.90 72.60	University students face heightened mental health challenges exacerbated by COVID-19. We surveyed Lebanese students and developed ML models to predict depression, anxiety, and stress. Our aim is to enable early intervention and tailored support for students' mental well-being.
					LR (D) (A)	-	64.0 74.0	74.12 74.89	
					ADABOO ST(D) (A)	-	68.0 69.0	76.25 74.89	
					RF(D) (A)	-	67.0 66.0	78.22 69.93	
					XGBOOST (D) (A)	-	68.0 68.0	75.55 6767	
					SVM(D) (A)	-	66.0 72.0	74.36 72.37	
					NB(D) (A)	-	65.0 76.0	74.12 76.37	
					KNN(D) (A)	-	46.0 62.0	66.63 61.05	
[45]	DA	College students during the Argentinean COVID-19 quarantine period.	Categorica l data	2,687	LR	0.78	0.73	0.90	To construct and evaluate machine learning (ML) algorithms aimed at forecasting depression levels among Argentinean students amidst the pandemic. To evaluate the effectiveness of classification and regression models by employing relevant performance metrics. To pinpoint the crucial features influencing the prediction of depression.
					RF	0.80	0.80	0.92	
					SVM	0.77	0.72	0.90	
[46]	DA	Social media posts from platforms (Twitter, facial expression databases,	Image Video text		LR	0.98	0.98	0.91	It highlights the importance of removing the stigma around mental health and the role of technology in providing support and interventions.
					RIDGE	0.98	0.98	0.8	
					DT	0.79	0.78	0.74	
					NB	0.67	0.75	0.64	
				2,500,000					
					LSTM	0.9	0.94	0.94	
[47]	DA	3000 Tweets	Text- processing and Twitter Sentiment Analysis	3,000	DT	-	0.82	0.482	Various ML-based approaches are exploited to find whether a Twitter user is depressed or not based on his/her social network behavior and tweets.
					NB	-	0.461	0.461	
					SVM-L	-	0.558	0.558	
					SVM-K	-	0.650	0.650	
[48]	DA	Health Behaviors School Children questionnaire during the 2013-2014 academic year.	Categorica l data	3,984	DT(D) (A)	-	88.5 74.2	86.7 73.7	Early detection and intervention in the life of college students are crucial to mitigate the long-term consequences of these conditions. However, interventions are primarily focused on prevention or treatment rather than prediction and risk factors using machine learning.
					SVM(D) (A)	-	93.7 76.3	96.8 85.1	
					RF(D) (A)	-	93.3 78.5	97.2 86.8	
					ANN(D) (A)	-	92.3 75.7	96.8 84.0	
					NB(D) (A)	-	89.9 72.8	95.5 82.3	
[49]	DA	Two hundred eighty-four undergraduate students of Systems Engineering and Computer Science University in Peru (Using Generalized Anxiety Disorder Questionnaire (GAD-7))	Categorica l data	284	KNN	80.70	67.85	-	To detect and intervene early using machine learning to improve anxiety prediction, offering timely support.
					NB	87.72	79.17	-	
					DT	73.68	63.92	-	
					GB	89.49	63.68	-	
					SVM	96.49	91.68	-	
					SO-KNN	97.83	97.83	-	
					SO-SVM	97.83	97.88	-	
[50]	DA	5685 students in grades 5 to 9 (aged 10-15 years) from public schools administered by the Palestinian Authority and United Nations Relief and Works Agency (UNRWA) schools.	Categorica l data	294	DT (D) (A)	-	88.5 74.2	86.7 73.7	This study aims to predict depression and anxiety risk factors among Palestinian school children using machine learning, facilitating tailored prevention and intervention programs to enhance their mental health and cognitive development.
					SVM(D) (A)	-	93.7 76.8	96.8 82.1	
					RF(D) (A)	-	93.3 78.5	97.2 86.8	
					ANN(D)	-	92.3	96.8	
					(A)		75.7	84.0	
					RF(D) (A)	-	89.9 72.8	95.5 82.3	

**Note:** D= Depression, A= Anxiety, ADHD = Attention Deficit Hyperactivity Disorder, fMRI = functional Magnetic Resonance Imaging, s-MRI = structural Magnetic Resonance Imaging, CNN = Convolutional Neural Network, SVM = Support Vector Machine, NN = Neural Network, LR= Logistic Regression, KNN = k-Nearest Neighbor, DT = Decision Tree, NB = Naïve Baye, RF = Random Forest, EEG = Electroencephalography, NHS (SWYPFT) = National Health Service Specialist Mental Health Provider (South West Yorkshire Partnership NHS Foundation Trust, XGBoost = extreme gradient boosting, CANTAB = Cambridge Neuropsychological Test Automated Battery, LASSO = Least Absolute Shrinkage and Selection Operator, BERT = Bidirectional Encoder Representations from Transformers (BERT), SVM-K = Support Vector Machine with kernel method, MLP = Multi-Layer Perceptron, SMO = Sequential Minimal Optimization (used for training support vector machines), BN = Bayesian Network, KS = K-Support, RS = Random Subspace, J48 = C4.5 Decision Tree algorithm, RT = Regression Tree.

## Data Availability

All the data and supportive information are provided within the article.

## LIST OF ABBREVIATIONS

RF: Random Forest
SVM: Support Vector Machine
ML: Machine Learning
WHO: World Health Organization

## STANDARDS OF REPORTING

PRISMA guidelines and methodology were followed.

## SUPPLEMENTARY MATERIAL

PRISMA checklist is available as supplementary material on the publisher’s website along with the published article. Supplementary material is available on the publisher’s website along with the published article.
