# Supplementary material for: Machine Learning Techniques to Predict Mental Health Diagnoses: A Systematic Literature Review
Source: Clin Pract Epidemiol Ment Health. 2024 Jul 26;20:e17450179315688. doi: 10.2174/0117450179315688240607052117 (PMC11443461; doi:10.2174/0117450179315688240607052117)
Supplement: Supplementary file 1 [file CPEMH-20-E17450179315688_SD1.pdf]

Clin. Pract. Epidemiol. Ment. Health ISSN: 1745-0179
DOI: 10.2174/0117450179315688240607052117, 2024, 20, e17450179315688 1

SUPPLEMENTARY MATERIAL **OPEN ACCESS**

Machine Learning Techniques to Predict Mental Health Diagnoses: A Systematic Literature Review

Ujunwa Madudubambachu[1,*], Augustine Ukpebor[2] and Urenna Ihezue[3]

[1]*School of Computing Sciences and Computer Engineering, University of Southern Mississippi, Hattiesburg, Mississippi, United States of America*
[2]*Cyber Insights, Jackson, Mississippi, United States of America*
[3]*Department of Public Health, College of Nursing and Health Professions, University of Southern Mississippi, Hattiesburg Mississippi, United States of America*

© 2024 The Author(s). Published by Bentham Open.
This is an open access article distributed under the terms of the Creative Commons Attribution 4.0 International Public License (CC-BY 4.0), a copy of which is available at: https://creativecommons.org/licenses/by/4.0/legalcode. This license permits unrestricted use, distribution, and reproduction in any medium, provided the original author and source are credited.

*Address correspondence to this author at the School of Computing Sciences and Computer Engineering, University of Southern Mississippi, Hattiesburg, Mississippi, United States of America, Tel: 601-447-1309;
E-mail: Ujunwa.madudubambachu@usm.edu

Published: July 26, 2024

Cite as: Madudubambachu U, Ukpebor A, Ihezue U. Machine Learning Techniques to Predict Mental Health Diagnoses: A Systematic Literature Review. Clin Pract Epidemiol Ment Health, 2024; 20: e17450179315688.
http://dx.doi.org/10.2174/0117450179315688240607052117

Send Orders for Reprints to
reprints@benthamscience.net

Supplementary Table 1. The summary of machine learning algorithms, advantages, and limitations in diagnosing mental health [1].

Algorithm	Advantages	Limitation
Support Vector Machine (SVM)	• Effective in handling high-dimensional data.<br>• Effective in dealing with non-linear decision boundaries using kernel functions.<br>• Tends to generalize well to unseen data.<br>• Can handle datasets with a small number of samples.	• Computationally expensive for large datasets.<br>• Difficult to choose appropriate kernel functions and hyperparameters.<br>• Sensitivity to noise in the dataset.<br>• May be challenging to interpret the model.
Random Forest (RF):	• Effective in handling high-dimensional data.<br>• Effective in dealing with non-linear decision boundaries using kernel functions.<br>• Tends to generalize well to unseen data.<br>• Can handle datasets with a small number of samples.	• Computationally expensive for large datasets.<br>• Difficult to choose appropriate kernel functions and hyperparameters.<br>• Sensitivity to noise in the dataset.<br>• May be challenging to interpret the model.
Logistic Regression	• Simplicity and interpretability of the model.<br>• Efficient computation, even with large datasets.<br>• Provides probabilistic predictions.<br>• Can handle both binary and multi-class classification problems.	• Assumes a linear relationship between predictors and the log odds of the response.<br>• May not handle complex non-linear relationships well. Sensitive to outliers.
Convolution Neural Network (CNN):	• Can model complex non-linear relationships.<br>• Can handle large-scale datasets.<br>• Can learn hierarchical representations of data.<br>• Generalizes well to unseen data after proper training	• Requires a large amount of data for effective training.<br>• Computationally expensive, especially for deep architectures.<br>• Prone to overfitting, particularly with small datasets.<br>• Difficult to interpret the learned representations.
Decision Tree	• Easy to understand and interpret.<br>• Can handle both numerical and categorical data.<br>• Can capture non-linear relationships and interactions.<br>• Does not require feature scaling.	• Prone to overfitting, particularly with complex trees.<br>• Sensitive to small changes in the data, leading to different tree structures.<br>• Limited generalization ability.<br>• Can create biased trees based on imbalanced data.

Algorithm	Advantages	Limitation
Naive Bayes (NB)	• Simple and computationally efficient.<br>• Effective with high-dimensional data.<br>• Robust to irrelevant features.<br>• Handles missing data well.	• Assumes independence between features.<br>• May not handle complex relationships between predictors well.<br>• Sensitivity to rare feature combinations.<br>• Tendency to be biased towards the majority class in imbalanced datasets
K-nearest neighbors (KNN)	• Simple and intuitive.<br>• Does not require training time.<br>• Effective in handling multi-class classification problems.<br>• Can handle noisy data.	• Computationally expensive for large datasets.<br>• Sensitive to the choice of distance metric and the value of K.<br>• Requires feature scaling for optimal performance.<br>• Requires storage of the entire dataset for predictions
Gradient Boosting Machine (GBM)	• Can handle diverse data types (numerical, categorical) effectively.<br>• Combines weak learners to create a strong predictive model.<br>• Robust to outliers and noise.<br>• Provides feature important rankings.	• Computationally expensive, especially for large datasets.<br>• Sensitive to overfitting, particularly with deep trees
Multi-layer Perceptron (MLP):	• Can model complex non-linear relationships.<br>• Can handle large-scale datasets.<br>• Can learn hierarchical representations of data.<br>• Effective in solving a wide range of machine-learning tasks.	• Requires careful tuning of hyperparameters.<br>• Prone to overfitting, especially with small datasets.<br>• Can be sensitive to feature scaling.<br>• Difficult to interpret the learned representations.

PRISMA 2020 CHECKLIST

Section and Topic	Item #	Checklist Item	Location where Item is Reported
**TITLE**			-
Title	1	Identify the report as a systematic review.	Title
**ABSTRACT**			-
Abstract	2	See the PRISMA 2020 for Abstracts checklist.	3
**INTRODUCTION**			-
Rationale	3	Describe the rationale for the review in the context of existing knowledge.	5 - 7
Objectives	4	Provide an explicit statement of the objective(s) or question(s) the review addresses.	72 - 94
**METHODS**			-
Eligibility criteria	5	Specify the inclusion and exclusion criteria for the review and how studies were grouped for the syntheses.	169 - 176, 181 - 185,<br>189 - 195
Information sources	6	Specify all databases, registers, websites, organisations, reference lists and other sources searched or consulted to identify studies. Specify the date when each source was last searched or consulted.	242
Search strategy	7	Present the full search strategies for all databases, registers and websites, including any filters and limits used.	178
Selection process	8	Specify the methods used to decide whether a study met the inclusion criteria of the review, including how many reviewers screened each record and each report retrieved, whether they worked independently, and if applicable, details of automation tools used in the process.	178
Data collection process	9	Specify the methods used to collect data from reports, including how many reviewers collected data from each report, whether they worked independently, any processes for obtaining or confirming data from study investigators, and if applicable, details of automation tools used in the process.	178
Data items	10a	List and define all outcomes for which data were sought. Specify whether all results that were compatible with each outcome domain in each study were sought (*e.g.* for all measures, time points, analyses), and if not, the methods used to decide which results to collect.	242
	10b	List and define all other variables for which data were sought (*e.g.* participant and intervention characteristics, funding sources). Describe any assumptions made about any missing or unclear information.	242
Study risk of bias assessment	11	Specify the methods used to assess risk of bias in the included studies, including details of the tool(s) used, how many reviewers assessed each study and whether they worked independently, and if applicable, details of automation tools used in the process.	178
Effect measures	12	Specify for each outcome the effect measure(s) (*e.g.* risk ratio, mean difference) used in the synthesis or presentation of results.	-

Section and Topic	Item #	Checklist Item	Location where Item is Reported
Synthesis methods	13a	Describe the processes used to decide which studies were eligible for each synthesis (*e.g.* tabulating the study intervention characteristics and comparing against the planned groups for each synthesis (item #5)).	175
	13b	Describe any methods required to prepare the data for presentation or synthesis, such as handling of missing summary statistics, or data conversions.	-
	13c	Describe any methods used to tabulate or visually display results of individual studies and syntheses.	207
	13d	Describe any methods used to synthesize results and provide a rationale for the choice(s). If meta-analysis was performed, describe the model(s), method(s) to identify the presence and extent of statistical heterogeneity, and software package(s) used.	-
	13e	Describe any methods used to explore possible causes of heterogeneity among study results (*e.g.* subgroup analysis, meta-regression).	-
	13f	Describe any sensitivity analyses conducted to assess robustness of the synthesized results.	-
Reporting bias assessment	14	Describe any methods used to assess risk of bias due to missing results in a synthesis (arising from reporting biases).	-
Certainty assessment	15	Describe any methods used to assess certainty (or confidence) in the body of evidence for an outcome.	-
**RESULTS**			-
Study selection	16a	Describe the results of the search and selection process, from the number of records identified in the search to the number of studies included in the review, ideally using a flow diagram.	235 - 241
	16b	Cite studies that might appear to meet the inclusion criteria, but which were excluded, and explain why they were excluded.	189 - 195
Study characteristics	17	Cite each included study and present its characteristics.	187 - 195
Risk of bias in studies	18	Present assessments of risk of bias for each included study.	187 - 195
Results of individual studies	19	For all outcomes, present, for each study: (a) summary statistics for each group (where appropriate) and (b) an effect estimate and its precision (*e.g.* confidence/credible interval), ideally using structured tables or plots.	187-195
Results of syntheses	20a	For each synthesis, briefly summarise the characteristics and risk of bias among contributing studies.	187-195
	20b	Present results of all statistical syntheses conducted. If meta-analysis was done, present for each the summary estimate and its precision (*e.g.* confidence/credible interval) and measures of statistical heterogeneity. If comparing groups, describe the direction of the effect.	187 - 195
	20c	Present results of all investigations of possible causes of heterogeneity among study results.	187 - 195
	20d	Present results of all sensitivity analyses conducted to assess the robustness of the synthesized results.	187 - 195
Reporting biases	21	Present assessments of risk of bias due to missing results (arising from reporting biases) for each synthesis assessed.	187 - 195
Certainty of evidence	22	Present assessments of certainty (or confidence) in the body of evidence for each outcome assessed.	187 - 195
**DISCUSSION**			-
Discussion	23a	Provide a general interpretation of the results in the context of other evidence.	72
	23b	Discuss any limitations of the evidence included in the review.	73 - 79
	23c	Discuss any limitations of the review processes used.	73 - 79
	23d	Discuss implications of the results for practice, policy, and future research.	73 - 79
**OTHER INFORMATION**			-
Registration and protocol	24a	Provide registration information for the review, including register name and registration number, or state that the review was not registered.	215 - 217
	24b	Indicate where the review protocol can be accessed, or state that a protocol was not prepared.	215 - 217
	24c	Describe and explain any amendments to information provided at registration or in the protocol.	215 - 217
Support	25	Describe sources of financial or non-financial support for the review, and the role of the funders or sponsors in the review.	215 - 217
Competing interests	26	Declare any competing interests of review authors.	215 - 217
Availability of data, code and other materials	27	Report which of the following are publicly available and where they can be found: template data collection forms; data extracted from included studies; data used for all analyses; analytic code; any other materials used in the review.	242

From: Page MJ, McKenzie JE, Bossuyt PM, Boutron I, Hoffmann TC, Mulrow CD, *et al*. The PRISMA 2020 statement: an updated guideline for reporting systematic reviews. BMJ 2021;372:n71. doi: 10.1136/bmj.n71
For more information, visit: http://www.prisma-statement.org/
